# A novel model of care for simplified testing of HBV in African communities during the COVID-19 pandemic in Spain

**DOI:** 10.1038/s41598-021-96350-3

**Published:** 2021-08-25

**Authors:** Camila A. Picchio, Daniel K. Nomah, Silvia G. Araujo, Ariadna Rando-Segura, Emma Fernández, Maria Buti, Sergio Rodríguez-Tajes, Sabela Lens, Francisco Rodríguez-Frías, Jeffrey V. Lazarus

**Affiliations:** 1 Barcelona Institute for Global Health (ISGlobal), Hospital Clínic, University of Barcelona, Barcelona, Spain; 2 Department of Health, Center for Epidemiological Studies on Sexually Transmitted Infections and HIV/AIDS in Catalonia (CEEISCAT), Generalitat of Catalonia, Badalona, Spain; 3 Liver Pathology Unit, Biochemistry and Microbiology Service, Hospital Universitari Vall d’Hebron, Barcelona, Spain; 4 CIBER Hepatic and Digestive Diseases (CIBERehd), Instituto Carlos III, Madrid, Spain; 5 Liver Unit, Hospital Universitari Vall d’Hebron, Barcelona, Spain; 6 Liver Unit, Hospital Clínic de Barcelona, IDIBAPS, Barcelona, Spain

**Keywords:** Epidemiology, Viral hepatitis, Health services

## Abstract

Chronic hepatitis B virus (HBV) infection is a major public health threat for migrant populations in Spain and efforts to scale up testing are needed to reach the WHO elimination targets. The Hepatitis B Virus Community Screening and Vaccination in Africans (HBV-COMSAVA) study aims to use point-of-care testing and simplified diagnostic tools to identify, link to care, or vaccinate African migrants in Barcelona during the COVID-19 pandemic. From 21/11/20 to 03/07/2021, 314 study participants were offered HBV screening in a community clinic. Rapid tests for HBsAg screening were used and blood samples were collected with plasma separation cards. Patients received results and were offered: linkage to specialist care; post-test counselling; or HBV vaccination in situ*.* Sociodemographic and clinical history were collected and descriptive statistics were utilized. 274 patients were included and 210 (76.6%) returned to receive results. The HBsAg prevalence was 9.9% and 33.2% of people had evidence of past resolved infection. Overall, 133 required vaccination, followed by post-test counselling (n = 114), and linkage to a specialist (n = 27). Despite the COVID-19 pandemic, by employing a community-based model of care utilizing novel simplified diagnostic tools, HBV-COMSAVA demonstrated that it was possible to diagnose, link to care, and vaccinate African migrants in community-based settings.

## Introduction

If left untreated, chronic hepatitis B virus (HBV) infection can lead to serious liver complications, such as cirrhosis or liver cancer^1^. Currently, an estimated two billion people worldwide have evidence of present or past HBV infection^2^, and estimates further report that 257 million people are currently chronic HBV carriers, of which the majority are living in sub-Saharan Africa (SSA) and South-East Asia^3^. Hepatitis B and hepatitis C virus (HCV) are responsible for more than 50% of all liver cancer cases, which is the leading cancer cause of mortality globally and the second on the African continent^4^. In West Africa, hepatocellular carcinoma (HCC) is the leading cause of cancer deaths in males and estimates report that 25% of young adults which have been infected during childhood will die prematurely from chronic HBV complications^5^.

In Spain, the number of migrants arriving from African countries such as Ghana has increased substantially in the last years, and the prevalence of HBV among these migrant groups needs to be addressed by the Spanish public health system^6^. A systematic review and meta-analysis in Ghana reported that the hepatitis B surface antigen (HBsAg) prevalence ranged from 0.55 to 14.3%^7^ and the risk of being infected with HBV in most sub-Saharan African countries is estimated to be more than 60%^8,9^. Given the high prevalence of HBV coupled with the elevated risk of infection acquisition and implementation of the HBV vaccine into the Expanded Programme on Immunization (EPI) only in 2002^10^, Ghanaian migrants in Spain are at increased risk of chronic HBV infection and late presentation to viral hepatitis care^11^. The prevalence of HBsAg in Catalonia, where Barcelona is, is estimated at 0.52%^12^, and HBV is treated and managed at the specialist level, which requires a referral pathway from primary care or another speciality. Current pathways to access HBV care in Spain remain complex and oftentimes unmanageable for migrant populations, particularly for those with irregular migratory status. Subsequently, health service use is lower among migrant populations as compared to the citizens of their host country^13^. In order to eliminate viral hepatitis in Europe, improving care for migrant populations is crucial^14^.

This underutilization of health services can result in late presentation to hepatitis care^15^, leading to delayed treatment initiation and ensuing liver damage progression, including irreversible liver cancer^16^. Point-of-care (PoC) testing and simplified diagnostic tools can facilitate screening and linkage to care to increase engagement and retainment in care^17^.

The aim of the Hepatitis B Virus Community Screening and Vaccination in Africans (HBV-COMSAVA) study is to use PoC diagnostic tools in community settings to identify and link to care or vaccinate African migrants in the greater Barcelona area in Catalonia, Spain.

## Methodology

This is a prospective cohort study (November 2020–January 2022) of adults (≥ 18 years) participating in a West African migrant community-based HBV screening, linkage to care, and vaccination program in the greater Barcelona area, Catalonia, Spain.

Preliminary data of the first seven months of the study (between 21 November 2020 and 3 July 2021) are reported. People attending religious services or community events were offered HBV screening in a “pop-up” clinic model of care (MoC) utilizing a combination of rapid detection tests (RDT) and simplified sample collection strategies. Participating centers were west African migrant faith-based and community organizations in the greater Barcelona area. Measures to reduce the spread of COVID-19 were in place during clinic hours, including mandatory facemask use at all times, hand sanitizing, and physical distancing. People currently on HBV treatment were excluded from the study.

### Intervention

On a previously agreed date, the study team would arrive and set up the clinic according to the available space and resources on intervention days (N = 20). Eleven sociodemographic items and twelve clinical history questions were collected verbally and reported in each patient data collection sheet. Trained culturally targeted peer navigators provided education on viral hepatitis prior to the screening and the team together answered all questions from participants. Pre-intervention training included an overview of the study and its objectives, an overview of HBV infection including modes of transmission and prevention. This approach was employed as it has proven vital in other viral hepatitis community-based intervention programs for migrants^18,19^.

Individuals who agreed to participate were offered a RDT (DETERMINE™ HBsAg 2, Abbott Laboratories) to screen for the presence of the HBV surface antigen (HBsAg) and also had a blood sample collected utilizing a plasma separation card (cobas^®^ plasma separation card (PSC), Roche Diagnostics), which required 140 µl of blood per spot (total of 3 spots) for analyzing HBV viral load (HBV-DNA) and co-infection with hepatitis D virus (HDV) through the presence of HDV antibodies (anti-HDV) if HBsAg+, or HBV core antibodies (anti-HBc) if HBsAg− (Box 1). Participants were given the option of sample collection via capillary blood from a fingerstick or whole blood collected intravenously. PSCs were left to dry and later transported to Vall d’Hebron University Hospital Laboratory (Barcelona) for analysis 1–5 days after being collected. Sensitivity and specificity of the PSC are very high (> 95%) and previously described^20^.

**Box 1:** Decision algorithm for care in the HBV-COMSAVA study, November 2020–July 2021.

HBsAg+	Refer to specialist care^
HBsAg−/anti-HBc+	Past resolved infection, inform of status and provide post-test counselling
HBsAg−/anti-HBc−	If no prior vaccination reported, offer vaccination against HBV

^Those who were HBsAg + were referred to a specialist irrespective of the HBV-DNA viral load detected on the PSC.

Participants were informed of the day the HBV-COMSAVA team would return to the same participating center to provide the results of the laboratory analyses and were also given a “return-date” reminder slip. If a person was HBsAg+, a referral to a specialist was provided during the first visit. Patients had the option of choosing between two university hospitals (Hospital Clínic of Barcelona and Hospital Vall d’Hebron) and were seen through an expedited referral process which did not require a prior appointment. Confirmation of diagnosis and treatment eligibility were determined by the hepatologists within the hospital setting and pathway. If a person was HBsAg− and did not have evidence of immunity, the first dose of the HBV vaccine (ENGERIX-B, GlaxoSmithKline Biologicals) was offered in situ free of charge, in addition to a referral for the subsequent doses at their corresponding primary care centers. The patient identifying code (CIP) for the public health system was recorded in order to document these vaccinations in the central health data system. Those who were anti-HBc+ or who reported prior HBV immunization were offered post-test counseling, which included an explanation of their serostatus and HBV awareness.

### Access to the public health system

Participants that did not already have a CatSalut health card (documentation that provides access to the Catalan public health system for residents of Catalonia) were offered an expedited processing system to facilitate its obtention. Requirements for inclusion in this expedited pathway included: state of vulnerability, inability to register their address, and health care need. This pathway was offered by the Red Cross of Catalonia in collaboration with the Health Department of Catalonia.

### Data collection

Data were collected from an epidemiological survey, the RDT result, and the laboratory reports. Data from each patient were collected and transferred to a non-person identifiable Excel sheet. Data were introduced using double-entry validation by two independent researchers, and doubts were resolved by examining the original survey results and laboratory reports.

### Variables

Sex, year of birth, country of birth and origin, year of arrival in Spain, pregnancy (for women), number of children, education level, and employment status were collected. Information regarding HBV vaccination status, ever being tested for HBV, having undergone surgery in Spain or in another country (if it was performed in a country other than Spain it was specified where), having a family member with HBV and whether that the HBV-positive individual lived in the same household and having a mother with a positive HBV diagnosis were collected as “yes/no/not sure”.

Other variables of interest included; having travelled to the African continent in the last year or planning to travel in the next 12 months, having been incarcerated, having engaged in risky situations in the past 6 months (multiple sexual partners, drug use, unprotected sexual encounter), having an STI or HIV diagnosis, having received a transplant, having an inflammatory bowel disease, having chronic renal insufficiency or receiving hemodialysis treatment, having any tattoos or scarring, and having any other illnesses and taking any medication.

### Data analysis

The prevalence of HBV infection was calculated for the whole sample and later stratified by sex, level of education, and years since arrival in Spain. Patients who were HBsAg+ were compared to those who were not. Mean, standard deviation and Student’s t-test were used for quantitative variables and frequency and chi-squared test were used for qualitative variables where the level of significance was set at < 0.05. Data were analyzed using StataCorp statistical software: Release 16^21^.

### Ethical considerations

This study received ethical clearance in 2020 from the Ethical Committee of the Hospital Clínic de Barcelona, Barcelona, Spain (n. HCB/2020/1036). This study was performed in accordance with relevant guidelines and regulations. All participants provided informed written consent. Study information sheets and informed consent forms were available in Spanish and English.

## Results

From 21 November 2020 to 3 July 2021, 314 people were offered an HBV test. Participants who have their second return visit programmed for a future date and have not yet received results (n = 34) are excluded from analysis. After removing participants who did not meet inclusion criteria (n = 6)**,** 274 were included. Reasons for exclusion included currently in care and receiving HBV treatment (n = 3) and not having an RDT nor dried plasma spot (DPS) sample collected (n = 3).

Women represented 42.3% (n = 116) of the sample, participants where predominantly of Ghanaian origin (92.0%, n = 252) and the mean age was 41.2 years (SD 10.3). Participants who reported (n = 86), on average, had 2.3 children (SD 1.4). The average number of years residing in Spain was 11.6 years (SD 6.5) with 27.0% (n = 74) of participants having arrived within the last five years.

### Hepatitis B infection

The overall prevalence of HBsAg+ detected utilizing the RDT was of 9.9%% (n = 27). Of these, eleven participants (40.7%) had detectable HBV-DNA viral loads, one (3.7%) did not have a PSC sample collected and was directly referred, and the remaining (55.6%, n = 15) were undetectable based on the PSC results. Two people (7.4%) had co-infection with HDV evidenced by the presence of anti-HDV on PSC. Prior resolved infection was evaluated in all those who were HBsAg− (n = 246) and was detected in 33.2% (n = 91) through the presence of anti-HBc. Sociodemographic variables and potential risk factors associated with HBV infection and anti-HBc positivity are described in Table 1.

**Table 1:** Baseline sociodemographic characteristics and risk factors of all patients screened and those who are HBsAg and anti-HBc positive.

	Overall N = 274 (%)	HBsAg+ n = 27 (%)	Anti-HBc+ n = 91 (%)
Mean age (SD)	41.2 (10.3)	41.4 (8.9)	42.3 (9.6)
Women	116 (42.3)	9 (33.3)	44 (48.3)
**Years of residence in Spain**			
1–5	74 (27.0)	8 (29.6)	25 (27.5)
6–12	61 (20.3)	7 (25.9)	21 (23.0)
13–20	116 (42.3)	10 (37.0)	38 (41.8)
21+	19 (6.9)	1 (3.7)	6 (6.6)
No data available	4 (1.5)	1 (3.7)	1 (1.1)
**Country of birth**			
Ghana	252 (91.9)	25 (92.6)	86 (94.5)
Senegal	15 (5.5)	1 (3.7)	5 (5.5)
Cameroon	2 (0.7)	0 (0.0)	0 (0.0)
Nigeria	1 (0.4)	0 (0.0)	0 (0.0)
Niger	1 (0.4)	0 (0)	0 (0)
Mali	1 (0.4)	0 (0)	0 (0)
Liberia	1 (0.4)	1 (3.7)	0 (0.0)
United Kingdom	1 (0.4)	0 (0.0)	0 (0.0)
**Level of education**			
No schooling	16 (5.8)	3 (11.1)	6 (6.6)
Primary school	43 (15.7)	5 (18.5)	12 (13.2)
Secondary school	173 (63.1)	14 (51.8)	67 (73.6)
University (bachelor, diploma)	23 (8.4)	2 (7.4)	2 (2.2)
Vocational training	10 (3.7)	1 (3.7)	3(3.3)
University (master or higher)	9 (3.3)	2 (7.4)	1 (1.1)
**Ever undergone surgery**	79 (30.8)	12 (44.4)	25 (27.5)
Of which were not in Spain	31 (39.2)	2 (16.7)	8 (32.0)
**Country where surgery was reported to have taken place**			
Ghana	26 (83.9)	2 (100)	6 (100)
Senegal	2 (6.4)	0 (0.0)	0 (0.0)
Nigeria	1 (3.2)	0 (0.0)	0 (0.0)
Russia	1 (3.2)	0 (0.0)	0 (0.0)
United Kingdom	1 (3.2)	0 (0.0)	0 (0.0)
STI diagnosis	4 (1.5)	1 (3.7)	2 (2.2)
Family member HBsAg+	23 (8.4)	4 (14.8)	9 (9.9)
Mother HBsAg+	3 (13.0)	2 (50.0)	0 (0.0)
HBsAg+ family member living in same household	8 (34.8)	0 (0.0)	3 (33.3)
Recent travel to Africa (≤ 6 months) or plans to travel in next 12 months	136 (49.6)	15 (55.6)	55 (60.4)
Tattoos or scarring	3 (1.1)	0 (0)	3 (3.3)
Ever been incarcerated	4 (1.5)	0 (0)	1 (1.1)

Men were twice as likely to have HBsAg positivity in comparison to women (18; 11.4% vs. 9; 7.8% respectively (*p* = 0.314)), while the positivity rate by age was almost the same for those who were HBsAg positive and negative (41.4 years vs. 41.2 years, respectively (*p* = 0.953)). Both differences in sex and age were statistically insignificant. Those who were HBsAg+ had been living in Spain, on average, for a shorter period of time as compared to those who were HBsAg− (10.6 years (SD 6.0) vs. 11.8 years (SD 6.6) (*p* = 0.376) and was not statistically significant. Education level was significantly associated with HBsAg positivity (*p* < 0.001) and both groups primarily completed up to secondary school (81.5% who are HBsAg positive v. 85.4% of those who are HBsAg negative).

### HBV vaccination

Most of the participants 192/274; 70.1%) reported not being vaccinated against HBV followed by not being sure (40/274; 14.6%). The remaining participants reported being vaccinated (37/247; 13.5%) or having one or two out of three doses of the vaccine (5/274; 1.8%).

### Cascade of care

**Figure 1:**
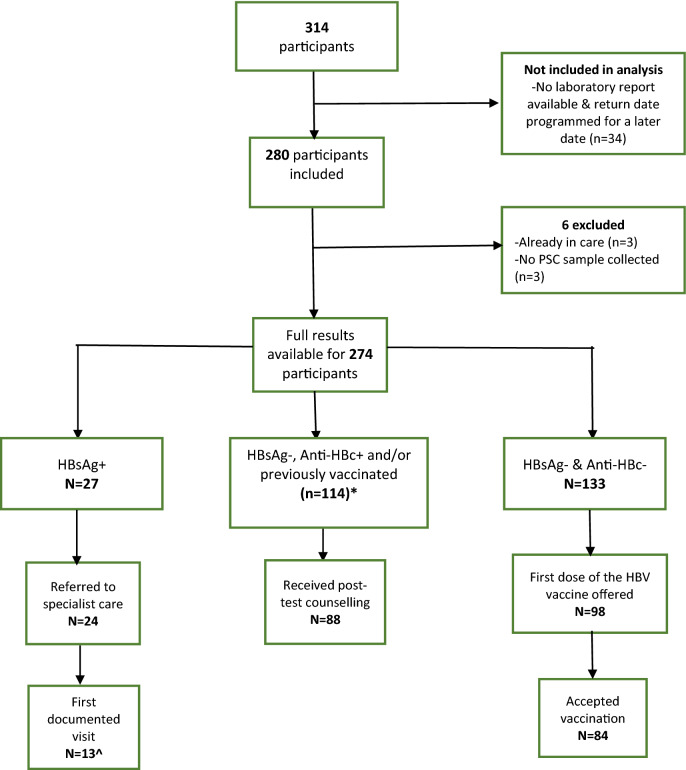
Flow chart of participants in the HBV-COMSAVA study and overall linkage to care proportions. *Fourteen participants reported being previously vaccinated and were anti-HBc positive. ^16 accepted the direct referral to one of the two hospitals linked to the project, of which 12 had a documented first visit with a specialist. Eight participants preferred visiting their own physicians (not the hospitals included in the study) due to distance and transportation issues, of which one had a documented first visit. *HBsAg* Hepatitis B surface antigen, *anti-HBc* antibody against hepatitis B virus core antigen, *HBV* hepatitis B virus.

Two hundred and ten (76.7%) participants returned to receive their results during the second community visit. The majority of patients (133; 48.5%) required vaccination, 114 (41.6%) were anti-HBc positive or previously vaccinated and required post-test counselling, and 27 (9.8%) were referred to a specialist (Table 2). Overall, the cascade of care for those who required referral or vaccination is described in Fig. 1.

**Table 2:** Patient treatment decisions and cascade of care proportions.

Patient treatment decisions	Total N	Number and % of patients who returned to receive results	Number and % of patients who accepted intervention among those who returned to receive results
**Post-test counselling**	114	88 (77.2%)	88 (100%)
Anti-HBc+	91*		
Self-reported past HBV vaccination	37*		
**Vaccination required**	133	98 (73.7%)	84 (85.7%)
**Referral to specialist care**	27	24 (88.9%)	13** (54.2.0%)

*14 participants reported being previously vaccinated and also were anti-HBc positive.

**16 accepted the direct referral to one of the two hospitals linked to the project, of which 12 had a documented first visit with a specialist. Eight participants preferred visiting their own physicians (not the hospitals included in the study) due to distance and transportation issues, of which one had a documented first visit.

### Accessing the Catalan health system

The majority of participants (266; 97.0%) reported having a CatSalut health card, which provides access to the Catalan public health care system. Eight participants (2.9%) requested expedited health insurance cards. Participants received their associated CIP via email and six received the physical card at their home during the project duration. The remaining participants are still waiting for the physical card to arrive but have access to the health system with the CIP number.

## Discussion

The COVID-19 pandemic has disrupted global efforts to curb the transmission of HBV and provide access to care. Difficult to reach and vulnerable populations, such as migrants, are particularly susceptible to these disruptions. Current reports show that about 90% of HBV services have been hampered by the current pandemic^22,23^. Prior to the COVID-19 pandemic, however, only approximately 5% of viral hepatitis cases were diagnosed globally^3^, and disruption to diagnostic capacity coupled with limited healthcare access due to over-burdened health systems may further impact HBV screening, treatment initiation and monitoring^23,24^. Missed diagnoses prevent adequate linkage to care and subsequent treatment initiation and monitoring. These missed opportunities can have long-term impact on transmission dynamics.

Despite the challenges due to the COVID-19 pandemic, by employing a novel dried plasma spot (DPS) test in combination with rapid detection testing, the HBV-COMSAVA model of care demonstrated that it was possible to diagnose, link to care, and vaccinate West African migrants in community-based settings during the pandemic who may otherwise not have received care. Therefore, in order to inform ongoing decision-making about how best to eliminate hepatitis B, preliminary results from this study are being made available.

The high prevalence of HBV infection reported by the HBV-COMSAVA study (9.9%) highlights the need to target interventions for this at-risk population. These results compare favorably with other studies from Spain^25^, and the United States^19^, which report prevalence rates of 9.6–33.6% among SSA migrants. In the European Union/European Economic Area (EU/EEA), the HBsAg prevalence among migrants from endemic countries is estimated at 6%, and between 1 and 1.9 million migrants born in endemic countries living in the EU are estimated to have chronic HBV infection^13^.

While HBV epidemiology varies by EU country, overall, the prevalence of HBV in the general population is around 1%^26,27^, and in Catalonia, Spain, the HBsAg estimated prevalence is 0.52%^12^. However, estimates report that migrant populations residing in Spain could have prevalence rates that are significantly greater in comparison^6,13^.

This significant difference in the rates between West African migrant populations and the Spanish general population reiterates the importance of making HBV prevention, diagnosis, and treatment services more accessible to this key population as (culturally and linguistically) tailored interventions.

More than 95% of respondents from 34 European countries reported decreased testing volumes for HIV, viral hepatitis, and STI during the COVID-19 pandemic^24^. This impact was reported in community services, non-governmental organizations, and in specialist care. Particularly affected were community services that provide care for key populations. Community‐based testing services, both fixed‐site and outreach‐based, represent potential sites to target specific population groups and individuals who may be at increased risk of infection and who are not in contact with formal health service^28^, as targeted screening campaigns tailored to the local demographics can better reach high-risk populations^17^.

The success of the HBV-COMSAVA model of care is, in part, due to its community-based nature since the team reaches a high prevalence population in settings which are comfortable and known to the people who the program is aimed to assist rather than waiting for patients to reach health care facilities. Evidence in Spain^29^, and other European countries^30^, has reported an underutilization of regular health care services by migrant populations, despite being accessible to them.

Diagnostic tools, like DPS sampling, can simplify the diagnostic process for difficult to reach populations and in resource-limited settings, and dried blood spot testing is recommended for use in these populations by the World Health Organization (WHO)^31^. The use of simplified diagnostic tools with high sensitivity and specificity are a key contribution to scaling up HBV testing. The PSC demonstrated a good concordance with standard serum/plasma samples as an alternative sample type for serological and virological testing used for screening of HBV and HDV infection. However, the limit of detection (LOD) of the techniques employed (serology and nucleic acid amplification tests (NAAT)) are affected by the lesser amount of starting material and dilution of the sample necessary for its reconstitution compared to plasma or serum. Therefore, the markers found in low concentration in plasma are difficult to detect. For this reason, this sample type has an excellent accuracy for the detection of chronically infected patients (HBsAg: sensitivity 97.0%, anti-HBc: sensitivity 97.0%) but low accuracy for the detection of resolved infection (anti-HBc: sensitivity 43.8%). This same situation occurs when we use NAAT tests. In HBeAg-negative patients, the sensitivity to detect HBV-DNA was low and can be explained by the low HBV-DNA titer of this kind of patients. However, this limitation would not have an impact on selection of patients eligible for HBV therapy; that is, those with HBV-DNA levels > 2000 IU/Ml. In this situation, the sensitivity of PSC increased to almost 96% and showed a high correlation with conventional venous blood testing^20^.

The HBV-COMSAVA model of care demonstrated how decentralized HBV testing in community-based settings can be another accessible care pathway for hepatitis care for at-risk vulnerable populations. The use of rapid detection testing and novel sample collection methods together with culturally trained peer navigators resulted in increased access to care, including referrals to specialist care or vaccination during the COVID-19 pandemic. This integrated approach can potentially be utilized for other health and social conditions affecting this population, such as non-communicable diseases like diabetes and hypertension^32^, as well as COVID-19 vaccination^33^.

Strategies to increase screening of and vaccination against HBV for migrant populations living in the EU/EEA have shown to be promising and cost-effective^34^, despite highlighted barriers to ensure effective strategies to link patients to care. While our study, overall, had high linkage-to-care proportions, loss to follow-up was reported for some participants requiring linkage to specialist care and vaccination. While positive cases are offered referrals during the first visit to limit patient drop-off, irrespective of their HBV viral load, sociocultural and economic factors such as work hours, transportation costs, and caregiving often times hinder successful linkage to care as evidenced in our ~ 50% documented first visit rate. However, our study shows that increased context-specific HBV testing among high-risk migrant populations utilizing novel simplified diagnostic tools is a suitable strategy to increase HBV diagnoses and prevent community-infection through vaccination.

## Limitations

The main limitation of our study includes the LOD of the PSC samples. The LOD of the techniques employed (serology and NAAT) are affected by the lower amount of starting material and dilution of the sample necessary for its reconstitution compared to plasma or serum. For this reason, some patients with low HBV DNA levels (< 1000 IU/mL) were not detected. On the other hand, some patients who were immune to hepatitis B due past resolved infection may not have been detected and were vaccinated unnecessarily. This, however, does not pose a risk to the patient. Additionally, our data may be affected by recall bias as participants may not have remembered whether they had received the HBV vaccination specifically or know whether family members are living with hepatitis B. We aimed to mitigate this by asking each participant if they had ever received three shots (a trait specific to this type of vaccine) of the HBV vaccine. Additionally, this is a cross-sectional cohort study so the results are descriptive in nature and the prevalence, for example, may not be generalisable to other West African migrant populations in Spain or elsewhere.

## Conclusions

This real-world demonstration launched during the COVID-19 pandemic showed that a model of care can screen, link to care, and vaccinate migrants for HBV in community settings and ensure access to care through the utilization of RDT and DPS testing. The study reports that close to 50% of the participants have current HBV infection or a previously resolved infection, which the majority of participants were previously unaware of. Additional studies should be carried out to better understand the barriers to accessing viral hepatitis care for migrants from different high-prevalence countries and ensure adequate linkage to care to prevent loss to follow-up and to better identify associated risk factors with HBV infection in those communities.
